# Advancing Chronic Disease Practice Through the CDC Data Modernization Initiative

**DOI:** 10.5888/pcd20.230120

**Published:** 2023-11-30

**Authors:** Timothy Jay Carney, Jennifer L. Wiltz, Kailah Davis, Peter A. Briss, Karen Hacker

**Affiliations:** 1 National Center for Chronic Disease Prevention and Health Promotion, Centers for Disease Control and Prevention, Atlanta, Georgia; 2 US Public Health Service, Atlanta, Georgia

Chronic disease affects 6 in 10 adults in the US, while 4 in 10 adults live with multiple chronic diseases (1). Chronic diseases represent one of the nation’s leading causes of disability and drivers of the nation’s $4.1 trillion in annual health care spending (1). Chronic conditions including heart disease, cancer, stroke, diabetes, and chronic kidney disease dominate the leading causes of death. Furthermore, leading lifestyle risk factors in the US include tobacco use, poor nutrition, physical inactivity, and excessive alcohol use (1).

Chronic disease prevention and control necessitates a comprehensive strategy to prevent disease (2–4), which is needed now more than ever (5). Information systems innovations are needed to advance health activities and outcomes and to allow decision makers and practitioners to act (4,6–8). Chronic disease data are a foundation that can inform interventions to promote healthy communities, support healthy behaviors and lifestyles, and facilitate effective and coordinated chronic disease prevention and health promotion (5,9). The benefits of an improved chronic disease data landscape include improved management of chronic disease programs, enhanced communication, data exchange, and coordination between federal, state, tribal, local, and territorial health departments and their partners. Additionally, efforts aimed at enhancing chronic disease surveillance practices will better enable a learning health system, precision public health, and improved situational awareness that will ultimately allow people across the US to live longer, healthier lives (10–12).

Despite the tremendous need for chronic disease data across all disease categories, gaps remain in the ability to rapidly translate data into action (7,8,13,14). Antiquated data systems need updating and modernization. This was particularly apparent during the COVID-19 pandemic when data flow between states and CDC was challenging (15,16). The lag time from initial chronic disease data capture to the availability of actionable information can be 2 years or more (17,18). Data are also often not available at the local level.

Data modernization efforts (19) are critical to building the capacity of the public health system to effectively monitor and address chronic disease. At the National Center for Chronic Disease Prevention and Health Promotion (NCCDPHP) within the Centers for Disease Control and Prevention (CDC), we aim to encourage better use of data and better meet the needs of our partners, more importantly ensuring the inclusion of those in health care, social services, and public health. The modernization activities as described in this manuscript have the goal of meeting the needs of the field and we hope that they are helpful. While we recognize that modernization will reflect a diverse portfolio of national, state, and local efforts crossing multiple sectors, this brief discussion will primarily focus on the federal response to chronic disease data challenges and how these efforts may represent and build support for greater, more comprehensive actions at all levels of public health.

## Understanding the Chronic Disease Data Challenges

To improve chronic disease data and address modernization efforts, we must account for the complexity in the chronic disease landscape and address the needs of 3 main partners: health care, social services, and public health. Each of these primary partners are essential to understanding chronic disease, each has unique data needs, and each possess their own chronic disease data. Challenges exist across these large-scale domains.

### Public health system

Public health collects population-based data as well as data on reportable conditions. It requires accurate, locally relevant, and timely data to provide comprehensive and pertinent information for decision-making. However, accessing these data is an ongoing challenge. Currently, much of this information comes from large population-based surveillance systems. These should be complemented with pipelines into clinical (eg, electronic health information), social service, jurisdictional health department (eg, population registries), community (eg, local community profiles), and other data sources that will allow improved evaluation of local risks and disease burden. Much of the data in these systems is publicly available but seldom connected to either the health care or the social service systems. Challenges, needs, and thus opportunity exist at the level of data (eg, population sampling, information missingness, data quality standards), information systems (eg, systems infrastructure, data management, information linkages, privacy protection), and contextual factors (eg, the policy landscape, payments, tools packaged for application) across the process of data capture, integration, and use (20).

### Health care system

Health care generates an enormous amount of individual-level chronic disease data necessary for care provision, billing, quality measures, and coordination of services. However, there continue to be barriers to accessing data and challenges to synthesizing data, in part because of fragmented systems. For those outside the health care system that could benefit from health care data, issues including privacy concerns, interoperability, and data quality all pose significant hurdles. While efforts are under way to improve access to health care data for public health use, to date no universal strategies exist to extract data from the health care system for public health uses.

### Social services system

Social services also collect data on clients and consumers but would benefit from better standardization and linkages to both health care and public health data sources. For example, social services may require data from the health care system for effective referral and follow up including information on the client’s social needs and support mechanisms. However, there is a lack of consistent case management systems that are interoperable with electronic health records. Information on measurable progress needs to be communicated to ensure quality management, but variability in systems leads to inconsistent definitions, quality control, and reporting. In addition, the social service system could benefit from data about upstream community conditions that contribute to population risk, but there are few examples of public health and social service data sharing that would meet this need. Major data gaps also exist in identifying populations with greatest needs, including populations who have lower socioeconomic status or education levels, persons living in sparsely populated rural areas, and those from racial and ethnic minority groups. Addressing data gaps in these areas should help detect, monitor, and manage chronic conditions and promote healthy populations.

### Across the chronic disease landscape

There is considerable data fragmentation within as well as between these silos. For example, the public health system at the national level maintains chronic disease data in multiple places. Some resources, like the Behavioral Risk Factor Surveillance System, cover a variety of chronic conditions. Others focus on a single chronic disease such as the Diabetes Surveillance System, the Paul Coverdell Stroke Registry, or the National Program of Cancer Registries. Similarly, within the health care system, there are countless registries and clinical data warehouses, health information exchanges, and claims databases that house and track data. Each data repository is typically managed by a different owner such as health care services, private companies, higher educational institutions, and government agencies. Some data are publicly available while others have limited access. All these disparate efforts result in fragmented views of the overall burden of chronic disease in the US within and across jurisdictions. They also result in challenges to generating up-to-date estimates of progress and shortfalls. While some progress has been made, there is no one model at the local or national level for chronic disease data modernization to adopt.

Addressing the challenges across these disparate systems will require solutions across the public health ecosystem. The CDC Moving Forward strategic initiative aims to modernize CDC to consistently deliver public health information in real time (21). The CDC Data Modernization Initiative (DMI) (22) intends to address many longstanding challenges of managing data and technology to support public health outcomes. While CDC activities will not apply seamlessly for all partners and will not address all challenges, we present the NCCDPHP portfolio of data modernization programs as good examples that might be more broadly reused or adapted.

## Highlighted Successes in Chronic Disease Modernization

CDC’s NCCDPHP is dedicated to improving the health and wellness of Americans by preventing and managing chronic disease. Data are a critical component of this mission. Modernizing approaches to generating and using chronic disease data will yield information to CDC, our partners, and the public in a timely, efficient, and accessible manner. NCCDPHP has launched efforts to modernize the chronic disease data landscape and now has a portfolio of over 27 projects. These projects touch various existing and new surveillance initiatives across all NCCDPHP operational units.

To better organize and coordinate the NCCDPHP data modernization effort, an NCCDPHP DMI strategic coordination plan was put in place. The activities were divided into 6 main areas that allowed for ease of tracking progress, reporting program updates, and promoting the value-added benefits to NCCDPHP program objectives. NCCDPHP data modernization priorities and activity areas (Table) include the following:

**Table:** Select Activities in the National Center for Chronic Disease Prevention and Health Promotion (NCCDPHP) Data Modernization Portfolio Across 6 Priorities and Diverse Health Topics^a^

NCCDPHP priorities	NCCDPHP portfolio highlights
1. Modernizing legacy information technology and surveillance systems	NCCDPHP is supplementing the Behavioral Risk Factor Surveillance System (BRFSS) data with internet panels to explore internet panels as an alternative to random population-based sampling. This project provided insight into whether and how to combine multiple data sets to refine population-based surveillance estimates.^b^
	NCCDPHP is building partnerships and leveraging National Center for Health Statistics infrastructure to improve interoperability between maternal and child health systems and vital records. For example, leveraging the State and Territorial Exchange of Vital Events (STEVE) for the real-time transmission of states’ vital records data into 3 surveillance systems — Pregnancy Mortality Surveillance System (PMSS), Pregnancy Risk Assessment Monitoring System (PRAMS), and Maternal Mortality Review Information Application (MMRIA, or Maria). This process reduces the burden of reporting on states and improves data efficiencies for CDC, which ultimately results in faster data dissemination.^c^
	NCCDPHP is incorporating new web-based survey modes of data collection within PRAMS and modifying the system to increase response rates, lessen burden, and improve the user experience. The modernization efforts will provide better data that will aid patients and health care providers in decision making and will provide information for developing, implementing, and evaluating programs and policies aimed at reducing health problems among mothers and babies.^b^
2. Using electronic health record platforms	NCCDPHP is using electronic health record (EHR) data from information networks to develop electronic phenotypes for disease conditions such as diabetes, nonalcoholic fatty liver disease, and hypertension, and to clean height and weight data to calculate more accurate estimates of body mass index. This work will support additional epidemiological studies.^d^
	NCCDPHP is piloting a distributed surveillance system, Multi-State EHR-Based Network for Disease Surveillance (23) to support timely access, transfer, and use (including on-demand analysis) of EHR data to produce estimates of chronic disease prevalences. Successes from this demonstration could lead to a near real-time surveillance system for monitoring trends, informing policies, planning programs, and evaluating outcomes.^b^
	NCCDPHP is using the Bidirectional Services e-Referral (BSeR) project to develop and implement national standards to enhance how information is exchanged between health systems and community service organizations who address chronic health conditions. For example, lifestyle change programs and diabetes self-management programs. This work will help health systems evaluate the impact of different community programs on people’s health. At the same time, it will help community-based organizations make the case for clinically meaningful and cost-effective programs.^d^^,^ e
3. Integrating data	NCCDPHP is leveraging cloud infrastructure to design center-wide data lake and data hub platforms that address end-to-end user needs. The NCCDPHP data lake will combine data resources and connect data across otherwise siloed data sets and systems. This will allow for cross-cutting data analysis, support novel data linkages, and allow for ever-increasing complexity in data queries. The data hub will manage large-scale data sets and allow for the generation of advanced analytics and data visualization, making reports and research more timely.^d^
	NCCDPHP’s National Diabetes Prevention Program (National DPP) Operations Center supports organizations offering the National DPP lifestyle change program and assists CDC staff in responding to questions and addressing programmatic challenges. It contains 2 applications: 1) the Recognized Organization Site Explorer, which provides summary statistics on CDC-recognized program delivery organizations, and 2) the Enrollment Tracker, which provides aggregate data on participants and their outcomes. Two other applications are in development: the Community Assets Map, which will help programs identify and collaborate with local community resources, and the Report Generator, which provides a means to collect, parse, and visualize data from the Diabetes Prevention Recognition Program database. The Operations Center was released to trained National DPP state quality specialists at the end of 2022; initial user feedback was positive, and continuing input will be collected to inform future site additions and modifications.^e^
	Data from 2 population-based surveys (BRFSS and National Health Interview Survey) were merged to assess the feasibility and potential benefits of merging data to refine population estimation of chronic conditions and risk factors at the state level. This resulted in estimates that have larger numbers of responses and decreased the margin of error in the estimation process.^b^
4. Leveraging cloud technologies	The Cancer Surveillance Cloud-Based Computing Platform (12) is a cloud platform that will ensure that data reporters can submit cancer data in real time and that will automate manual processes, which in turn makes cancer data available faster. This platform will provide a more up-to-date picture of cancer trends, which can inform cancer programs, interventions, policies, and treatment decisions to reduce the cancer burden in high-risk populations.^f^
	The Water Fluoridation Reporting System modernization project is seeking to modernize the online tool that helps states manage the quality of their water fluoridation programs and describes the percentage of the US population on community water systems that receives optimally fluoridated drinking water. The WFRS modernization will use cloud technology to enhance the system with additional functionality. The new system will provide more flexibility for data entry at the state and local levels, on-demand reporting and visualization, and easier data analysis.^g^
	Population Level Analysis and Community Estimates (24) provides model-based, population-level analysis and community estimates of health measures for all counties, incorporated and census designated places, census tracts, and zip code tabulation areas across the US. PLACES complements existing surveillance data by providing estimates necessary to understand health issues affecting local areas. The release of these data allows local health departments and jurisdictions, regardless of population size and rurality, to better understand the burden and geographic distribution of health issues in their areas and assist them in planning public health interventions.^b^
	Several maternal and child health surveillance systems (PRAMS, National Assisted Reproductive Technology Surveillance System [NASS], and MMRIA) are being or have been migrated to the cloud to automate data processing functions. The impact includes significantly reducing data delays and improving the quality of data made available to CDC and states related to pregnancy risks, maternal mortality, and other health effects.^c^
	The tobacco and smoking Media Campaign Resource Center is leveraging cloud-based technologies to increase the speed at which new features and enhancements are released to customers. The modernization efforts will accelerate the access to credible public information and materials on tobacco control. Partners accessing this resource will find support for their education and communication efforts.^h^
	The mobile app My Family Health Portrait: Cancer was developed to trace the illnesses suffered by one’s parents, grandparents, and other blood relatives, known as family health history. This information can help doctors predict the disorders to which someone may be at risk and help individuals take action to keep them and their family healthy. To ensure this information can be easily collected, stored, and shared, cloud and mobile technology were leveraged to a mobile app. Now, information on genes, habits, and environments is easily accessible via smartphones and can be shared with others or referenced during future health appointments. The mobile app empowers users, their family, and health care providers with knowledge and the ability to act on it.^i^
5. Using data to address health equity and social determinants of health challenges	The Social Determinants of Health Data Exchange for Chronic Disease Prevention Initiative is a collaborative effort to expand the collection, use, and exchange of social needs data from EHRs. The initiative developed SDOH business and use cases that that support safe electronic collection, aggregation, use, reuse, and reporting of SDOH data for chronic disease prevention and health promotion. The product from this work gives public and private decision makers a tool to extend proper data use and exchange beyond the health care visit and improve care coordination, use, and overall health for people and populations.^d^
	In another initiative that leveraged health equity and SDOH data for chronic disease, NCCDPHP acquired, accessed, and analyzed proprietary scanner data on retail sales of food, tobacco, alcohol, and suntan and sunscreen products by collaborating with relevant federal partner agencies (eg, US Department of Agriculture, Food and Drug Administration). Some analyses of the data included examination of 1) the nutritional quality of household food purchases, 2) the association between county-level food retail and socioeconomic environment, and 3) changes in nutritional quality of retail food sales in counties funded by the CDC High Obesity Program (HOP) to shed light on the effect of the program on county-level measures over time. Findings from these studies can be used to inform the development and implementation of effective and targeted interventions to support healthier dietary behaviors.^d^
	The Clinical and Community Data Initiative (25) links data from health care, community-based programs, and administrative data sets at the individual, household, and population level. The project team has built reusable tools to collect, combine, and query these data sources and are testing these in Colorado and North Carolina. Successes to date include strengthening connections between health systems and community-based organizations, generating insights for partnering organizations, and creating a geocoded, longitudinal data set with child obesity–related data from 3 large health care systems and 2 community organizations for end-user researchers in Denver, Colorado.^j^
	NCCDPHP expanded the PRAMS surveillance system to include an SDOH module to address gaps in the standard data collection of SDOH data on surveys. The module is implemented at 22 sites and includes questions on housing, food insecurity, transportation, and discrimination. The effort improves the data infrastructure and availability to support research and practice related to SDOH, maternal health, health equity, policy, and program evaluations.^c^
	NCCDPHP expanded BRFSS to include additional questions on SDOH equity. The questions capture data on topics such as racism, food and housing insecurity, transportation, social support, well-being, and economic stability.^b^
6. Building informatics planning and reporting capacity	NCCDPHP developed the Information Technology Strategic Planning and Reporting Tool to facilitate the rapid implementation of the CDC Data Modernization Initiative implementation plan.^d^

Abbreviation: CDC, Centers for Disease Control and Prevention.

a This table provides only a snapshot of NCCDPHP data modernization efforts across the Center’s divisions and offices; it is not a comprehensive list of data modernization activities.

b Division of Population Health.

c Division of Reproductive Health.

d NCCDPHP Office of the Director.

e Division of Diabetes Translation.

f Division for Heart Disease and Stroke Prevention.

g Division of Oral Health.

h Office on Smoking and Health.

i Division of Cancer Prevention and Control.

j Division of Nutrition, Physical Activity, and Obesity.

1. Modernizing legacy information technology and surveillance systems — NCCDPHP hosts close to 80 data systems and over 25 surveillance systems. Modernizing these systems is ongoing to increase data samples, connectivity, and usability.
2. Using electronic health record (EHR) platforms — Health care data largely stored in EHRs can be a source for health monitoring and quality of care improvements. Clean, accurate, timely, and complete EHR data could complement NCCDPHP surveillance efforts and support program activities.
3. Integrating data — Understanding and reducing chronic disease will require data across silos, bridges between disease data systems, and improvements in the flow of data. This will require large-scale data integration that can keep pace with modern query demands, produce integrated and dynamic analytics, support predictive capabilities, and produce efficiencies and economies of scale in design that provide better data for better results.
4. Leveraging cloud technologies — Cloud computing is a technological step forward that will allow enhanced data security, increased scalability, faster integration of data, automated reporting, and the promise of artificial intelligence computing that will enable dynamic processing of complex data queries and machine learning.
5. Using data to address health equity and social determinants of health (SDOH) challenges — Reducing health inequities and managing risk and vulnerabilities related to adverse SDOH is essential to improving chronic disease outcomes. Better data are needed to highlight areas of need, demonstrate what works, and allow for rapid strategy and intervention design to meet challenges as they arise.
6. Building informatics planning and reporting capacity — Strategic planning itself is a core practice supporting large-scale data modernization efforts. It allows planning for spending and investment in areas such as analytics reporting, dashboards, and related tools that are essential to see that desired results are being achieved.

The NCCDPHP modernization portfolio includes activities that impact the data lifecycle across the 6 priorities and address multiple chronic disease topical areas (Table). Select examples illustrate changes that might be useful:

- New information is being generated such as Population Level Analysis and Community Estimates (24) (PLACES; priority 4 in Table), that provides multiple health measures at local levels across the US. PLACES provides model-based estimates that can enhance understanding for localities, regardless of population size and rurality, of the burden and geographic distribution of health measures in their areas and assist them in planning public health interventions. In summer of 2024, a new SDOH module will also be available.
- Data are being linked across multiple sectors such as in the state-wide Clinical and Community Data Initiative (25) (priority 5 in Table). This project is linking data in North Carolina and Colorado across health care–based and community-based programs, and administrative data sets using common technology. Being able to link data on health behaviors, interventions, and outcomes to SDOH and other community services and factors provides more holistic views that are being used to reduce chronic diseases and achieve health equity (25).
- Complex data partnerships have been developed by the Multi-State EHR-Based Network for Disease Surveillance (23) (MENDS; priority 2 in Table). This network is helping to determine whether data routinely stored in EHRs can provide clinically detailed, efficient, and timely information from diverse populations both to answer pressing health questions and to yield reliable chronic disease estimates for public health surveillance. One example is from Washington, where MENDS data visualization tools are used to understand the distribution of hypertension and diabetes at a zip-code level (26).
- Data are being moved and stored securely, faster, and more flexibly through the use of new technologies. The National Program of Cancer Registries is transitioning to a newly designed cloud-based platform, the Cancer Surveillance Cloud-based Computing Platform (CS-CBCP; priority 4 in Table) (12). This move will automate cancer case collection processes, which will in turn make cancer data available in near real time. Similarly, a cloud data platform is being developed that improves data access and dissemination of the Pregnancy Risk Assessment Monitoring System (27) (PRAMS; priority 4 in Table) analytics research file. Expected benefits include lessening data access burden on partners and reducing data dissemination turnaround time (from 6 to 8 weeks to 2 or 3 days), ultimately reducing the time from data to action for mothers and children.
- Data are being translated into actionable information. One example is the National Diabetes Prevention Program (National DPP) Operations Center (28) (priority 3 in Table), which is a central data hub that provides critical and timely information and tools to CDC staff and organizations offering the National DPP lifestyle change program. These end users are now able to answer critical questions, solve programmatic challenges, and make data-driven decisions that can improve the delivery of and referral to the National DPP lifestyle change program. The use of the National DPP Operations Center may help scale and spread the National DPP to reach even more at-risk individuals (28).

## The Road Ahead for Chronic Disease Data Modernization

While examples of data modernization for chronic disease surveillance are under way nationally, more work is needed to use data resources to realize measurable impact on chronic disease outcomes. A comprehensive strategy for chronic disease modernization would address the inherent uniqueness of chronic disease surveillance across data types, collection methods, and systems at every level of public health. This must include activities such as improving data capture and quality for SDOH and demographic data, including race and ethnicity, to advance understanding of the drivers of health and equity; incorporating chronic disease within data flow pipelines; better supporting granular assessments of small segments of the population normally excluded in large aggregated estimates; reducing burden on partners engaged in surveillance activities; and building a skilled workforce to leverage advanced data practices and workflows. As we bolster the public health infrastructure after the pandemic, access to timely, accurate chronic disease data is foundational. Exemplars are already occurring in the field, and leaders in chronic disease can forge the path forward to better use data and information to improve the health of people across the US.
